# Predictive value of a reduction in the level of high-density lipoprotein-cholesterol in patients with non-small-cell lung cancer undergoing radical resection and adjuvant chemotherapy: a retrospective observational study

**DOI:** 10.1186/s12944-021-01538-1

**Published:** 2021-09-20

**Authors:** Fan Luo, Kang-mei Zeng, Jia-xin Cao, Ting Zhou, Su-xia Lin, Wen-juan Ma, Yun-peng Yang, Zhong-han Zhang, Fei-teng Lu, Yan Huang, Hong-yun Zhao, Li Zhang

**Affiliations:** 1 State Key Laboratory of Oncology in South China, Collaborative Innovation Center for Cancer Medicine, Department of Medical Oncology, Guangdong Esophageal Cancer Institute, Sun Yat- sen University Cancer Center, 510060 Guangzhou, People’s Republic of China; 2 State Key Laboratory of Oncology in South China, Collaborative Innovation Center for Cancer Medicine, Department of Pathology, Guangdong Esophageal Cancer Institute, Sun Yat-sen University Cancer Center, 510060 Guangzhou, People’s Republic of China; 3 State Key Laboratory of Oncology in South China, Collaborative Innovation Center for Cancer Medicine, Department of Clinical Research, Guangdong Esophageal Cancer Institute, Sun Yat- sen University Cancer Center, 510060 Guangzhou, People’s Republic of China

**Keywords:** Non-small-cell lung cancer, Adjuvant chemotherapy, High-density lipoprotein-cholesterol, Disease-free survival, Prognosis

## Abstract

**Background:**

Cancer patients often exhibit chemotherapy-associated changes in serum lipid profiles, however, their prognostic value before and after adjuvant chemotherapy on survival among non-small-cell lung cancer (NSCLC) patients is unknown.

**Methods:**

NSCLC patients undergoing radical resection and subsequent adjuvant chemotherapy from 2013 to 2017 at Sun Yat-sen University Cancer Center were retrospectively reviewed. Fasted serum lipid levels were measured before and after chemotherapy. The optimal lipid cut-off values at baseline and fluctuation were determined using X-tile™. The fluctuations in serum lipid levels and disease-free survival (DFS) were assessed.

**Results:**

Serum cholesterol, low-density lipoprotein cholesterol (LDL-C), high-density lipoprotein-cholesterol (HDL-C), triglyceride, apolipoprotein (Apo) A-I, and ApoB all significantly increased after adjuvant chemotherapy. X-tile determined 1.52 mmol/L of HDL-C and 0.74 g/L of ApoB as the optimal cut-off values before chemotherapy. Patients with HDL-C ≥ 1.52 mmol/L (median DFS: not reached vs. 26.30 months, *P* = 0.0005) and a decreased HDL-C level after adjuvant chemotherapy (median DFS: 80.43 vs. 26.12 months, *P* = 0.0204) had a longer DFS. An HDL-C level that increased by ≥ 0.32 mmol/L after chemotherapy indicated a worse DFS. A high baseline ApoB level were associated with a superior DFS. In the univariate analysis and the multivariate Cox analyses, a high baseline HDL-C level and a HDL-C reduction after adjuvant chemotherapy were independent indicators for superior DFS. High baseline HDL-C was related to N0-1 stage (χ^2^ = 6.413, *P* = 0.011), and HDL-C fluctuation was significantly correlated with specific chemotherapy regimens (χ^2^ = 5.002, *P* = 0.025).

**Conclusions:**

Adjuvant chemotherapy increased various lipid levels in resected NSCLC patients. A higher HDL-C level before chemotherapy and a reduced HDL-C level after adjuvant chemotherapy were independent predictors of longer DFS in patients with curable NSCLC.

## Background

Lung cancer (LC) is the most common cancer, with 80−85% of cases non-small-cell lung cancer (NSCLC) [1–3]. Almost one-third of NSCLC patients are candidates for resection [4, 5], and the number is increasing thanks to more and improved lung cancer screening [6]. However, even after curative resection, 10–75% of patients develop local or distant recurrence [7]. For stage II–IIIA patients undergoing radical resection, adjuvant chemotherapy is recommended as the standard of care to improve outcomes [8]. To date, the tumor, node, and metastasis staging system is the most discriminative indicator of prognostic value [7]. However, 5-year survival rates range from 73 % for stage IA disease to 13 % for stage IV disease, and even patients with the same stage can have heterogenous outcomes [7]. As such, more predictive methods are needed.

Several reports have demonstrated that aberrant metabolism of lipids can affect cancer development [9–11]. An altered serum lipid profile [cholesterol, low-density lipoprotein-cholesterol (LDL-C), high-density lipoprotein-cholesterol (HDL-C), apolipoprotein (Apo) A-I (the major protein component of HDL-C), ApoB (the main protein component of LDL-C), and triglyceride (TG)] is often observed in cancer patients. Dysfunctional lipid metabolism in NSCLC has also been reported. The total level of cholesterol (particularly HDL-C) tends to be inversely linked to LC risk [12, 13]. Similarly, ApoA-I and ApoB play a role in LC development. The ApoA-I serum level is inversely correlated with an increased risk of LC, whereas, ApoB is positively associated with LC incidence [14]. Whether the abnormal metabolism of lipids affects the prognosis of NSCLC patients receiving curative treatments is unknown.

Chemotherapy agents can also affect lipid metabolism. A few studies assessed breast cancer patients who received chemotherapy, and discovered significantly increased total cholesterol, LDL-C, and TG levels [15–18]. In colorectal cancer patients, chemotherapy significantly increased HDL-C and decreased LDL-C [19]. The molecular mechanisms underlying chemotherapy-related lipid imbalance are unclear. Additionally, cytotoxic drugs have been reported to influence system lipid metabolism by affecting cholesterol synthesis and efflux [20].

Chemotherapy-related lipid alteration can predict patients’ outcomes. When patients with acute lymphoblastic leukemia (ALL) were treated with induction treatment, dynamic increases in serum HDL-C and ApoA1 predicted better chemotherapy efficacy [21]. For patients with small cell lung cancer treated with chemotherapy, elevation in serum LDL-C predicted poor survival [22]. Yet, whether fluctuations in lipid levels exist in patients with NSCLC undergoing adjuvant chemotherapy, and whether they can predict NSCLC prognosis is unknown.

This retrospective study aimed to assess disease-free survival (DFS) prediction using dynamic lipid changes after adjuvant chemotherapy for NSCLC patients undergoing curative resection.

## Methods

### Patients

The inclusion criteria were: (a) pathologically confirmed primary NSCLC stage IB–IIIA; (b) available clinical information (sex, age, height, weight, smoking history, drinking history, diabetes mellitus, hyperlipidemia, metabolic syndrome, hypertension comorbidities, and the use of lipid-affecting drugs); (c) underwent chemotherapy for four to six cycles in an adjuvant setting following radical resection; (d) age ≥ 18 years old; (e) data available on fasted serum lipid profiles measured within 1 week before receiving the first chemotherapy cycle and between 2 to 4 weeks after the last chemotherapy treatment (cholesterol, TG, HDL-C, LDL-C, ApoA-I, and ApoB); (f) normal liver function (alanine aminotransferase (ALT), aspartate aminotransferase (AST), and total bilirubin (T-BIL)) measured within 1 week before starting adjuvant treatment.

The exclusion criteria were: (a) neoadjuvant chemoradiotherapy; (b) concomitant tumors or malignant tumors diagnosed within 3 years of the LC diagnosis; (c) use of drugs affecting lipid metabolism before serum collection (mainly statins); (d) concomitant diseases associated with lipid metabolism-related disease, such as diabetes, hyperlipidemia, or metabolic syndrome; (e) clinical obesity, a BMI ≥ 30 kg/m^2^; and (f) concomitant hypertension.

**Fig. 1:**
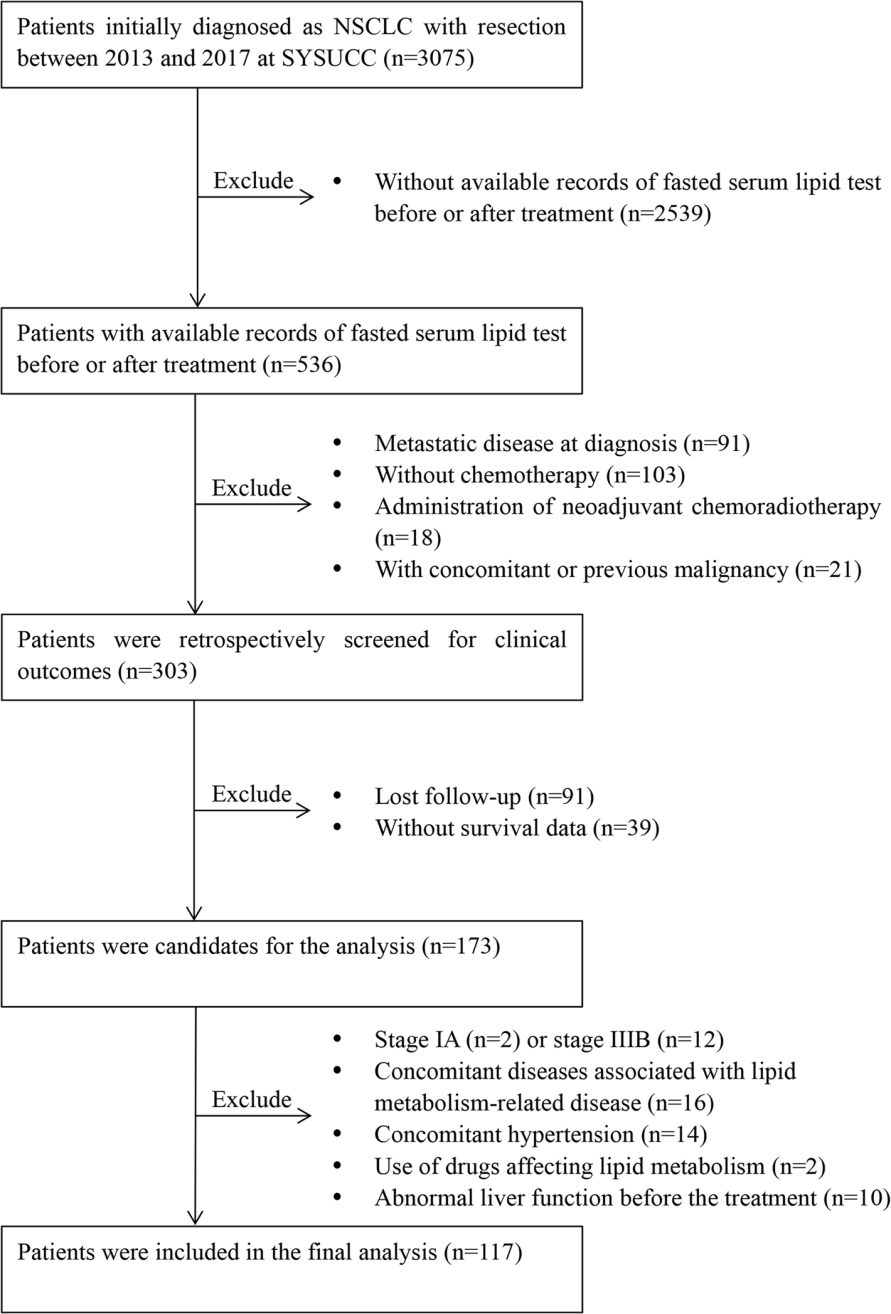
Study flowchart

Figure 1 shows the patient enrollment process. One hundred and seventeen patients were assessed with NSCLC who received curative-intent resection from 2013 to 2017 at Sun Yat-sen University Cancer Center (SYSUCC), Guangzhou, China. This study was conducted in accordance with the ethical standards of SYSUCC, and the Declaration of Helsinki (2013).

### X-tile™

X-tile (Yale University School of Medicine, New Haven, CT, USA) offers a rigorous statistical estimation for dividing a cohort into a “low” or “high” group for lipid serum levels. For DFS, X-tile plots were used to evaluate the optimal cutoff values for serum lipids according Camp et al.’s method [23].

### Data collection

As part of the physical examination, fasted serum lipid profiles were analyzed before and after adjuvant chemotherapy. Fasted blood specimens were collected before breakfast and tested using an automatic biochemical analyzer (Hitachi 7600-020, Japan).

Based on the medical records, demographic and clinical parameters (sex, age, height, weight, histology, smoking history, drinking history, diabetes mellitus, hyperlipidemia, metabolic syndrome, hypertension comorbidities, disease stage, radiotherapy, surgical type, adjuvant chemotherapy regimens, and use of lipid-affecting drugs) were collected. The tumor stage at the diagnosis of NSCLC was defined using the National Comprehensive Cancer Network guidelines.

The end-point of the study was DFS, which was defined as the period from surgery to the earliest radiographic evidence of recurrence, death, or the date of last patient access (censored). Radiological findings were confirmed by two experienced oncologists. Follow-up took place every 3 months for 2 years after surgery, and then every 6 months thereafter, or until death. The last follow-up was April 2020.

### Statistical analyses

Statistical analyses were performed using SPSS 25 (IBM, Armonk, NY, USA). Conventional statistics were used to study any fluctuations in the lipid levels and the correlations with clinical factors. The optimal cutoffs for serum lipid profiles were determined using X-tile, which helped to stratify patients according to different outcomes. The association between serum lipids with DFS was determined using the Kaplan–Meier survival methods. Cox proportional hazards regression models were applied for the univariate and multivariate analyses. Risk factors in the univariate analyses with a *P* value < 0.15 were included in the multivariate analyses. Data were the mean ± standard deviation (SD). Alterations in serum lipids and lipoproteins pre- and post-chemotherapy were compared using paired *t*-tests. A *P* < 0.05 was considered significant.

## Results

### Participants

Table 1 describes the clinical characteristics of this study’s cohort. The median follow-up was 58.8 months and patients were diagnosed at a median age of 56 (range, 31–70) years old. Most patients were male (69/117, 59 %). As for histology subtypes, lung adenocarcinoma accounted for 73.5 % (86/117) of cases. Fifty-four (46.2 %) cases were stage IB–II disease, and 63 (53.8 %) were stage IIIA disease. The main surgery type was lobectomy (93.3 %). Chemotherapy regimens administered mainly included cisplatin/carboplatin/nedaplatin + pemetrexed (86/117, 73.5 %). Other regimens were cisplatin/carboplatin/nedaplatin + docetaxel (14/117, 12.0 %), cisplatin/carboplatin/nedaplatin + paclitaxel (9/117, 7.7 %), and cisplatin + gemcitabine (8/117, 6.8 %).

**Table 1:** Characteristics of all patients

Characteristics	Cases (*n* = 117)	Percentage (%)
**Age (years)**		
Median (range)	56 (31–70)	
<65 vs. ≥65	99 vs. 18	84.6 vs. 15.4
**Gender**		
Male vs. female	69 vs. 48	59 vs. 41
**BMI**		
Median (range)	22.23 (16.42–29.39)	
< 24 kg/m^2^ vs. ≥24 kg/m^2^	79 vs. 38	67.5 vs. 32.5
**Smoking status**		
Non-smokers vs. smokers	63 vs. 54	53.8 vs.46.2
**Drinking status**		
Non-drinkers vs. drinkers	84 vs. 33	71.8 vs.28.2
**Histology**		
Adenocarcinoma vs. non-adenocarcinoma	86 vs.31	73.5 vs.26.5
**T stage**		
1–2	93	79.5
3–4	24	20.5
**N stage**		
0–1	62	53
2	55	47
**Disease stage**		
IB-II	54	46.2
IIIA	63	53.8
**Surgery type**		
Lobectomy	109	93.3
Pneumonectomy	8	6.7
**Radiotherapy**		
No	106	90.6
Yes	11	9.4
**Adjuvant chemotherapy regimens**		
Cisplatin/carboplatin/nedaplatin + pemetrexed	86	73.5
Others	31	26.5
Cisplatin/carboplatin/nedaplatin + docetaxel	14	12.0
Cisplatin/carboplatin/nedaplatin + paclitaxel	9	7.7
Cisplatin + gemcitabine	8	6.8

Abbreviations: *T *tumor, *N *node, *BMI *Body Mass Index

### Serum lipid and lipoprotein level comparisons before and after chemotherapy

The serum lipid profile before chemotherapy and after chemotherapy completion were analyzed for each patient to reveal any associations. The level of cholesterol, TG, HDL-C, LDL-C, ApoA-I, and ApoB all significantly increased after adjuvant chemotherapy (*P ≤* 0.001 for all) (Table 2). The mean HDL-C level pre- and post-chemotherapy was 1.20 mmol/L, and 1.44 mmol/L.

**Table 2:** Lipid alterations in all patients

Lipids	Mean ± SD	*P* value^ф^
**Cholesterol (mmol/L)**		**< 0.001**
Pre-chemotherapy level	4.73 ± 1.02	
Post-chemotherapy level	5.51 ± 1.00	
Difference^a^	0.78 ± 0.96	
**Triglyceride (mmol/L)**		**< 0.001**
Pre-chemotherapy level	1.22 ± 0.57	
Post-chemotherapy level	1.51 ± 0.81	
Difference^a^	0.29 ± 0.069	
**HDL-C (mmol/L)**		**< 0.001**
Pre-chemotherapy level	1.20 ± 0.32	
Post-chemotherapy level	1.44 ± 0.35	
Difference^a^	0.24 ± 0.36	
**LDL-C (mmol/L)**		**< 0.001**
Pre-chemotherapy level	2.98 ± 0.91	
Post-chemotherapy level	3.50 ± 0.88	
Difference^a^	0.52 ± 0.76	
**ApoA-I (g/L)**		**< 0.001**
Pre-chemotherapy level	1.17 ± 0.28	
Post-chemotherapy level	1.47 ± 0.27	
Difference^a^	0.30 ± 0.30	
**ApoB (g/L)**		**< 0.001**
Pre-chemotherapy level	0.98 ± 0.24	
Post-chemotherapy level	1.08 ± 0.24	
Difference^a^	0.10 ± 0.21	

Data are mean ± standard deviations

Abbreviations: *SD *standard deviations, *HDL-C *high-density lipoprotein cholesterol, *LDL-C *low-density lipoprotein cholesterol, *ApoA-I *apolipoprotein A-I, *ApoB *apolipoprotein B

^a^Difference = lipids post-chemotherapy –lipids pre-chemotherapy

^ф^Compared with paired t-test

### Serum lipid profile evaluation before chemotherapy using X-tile

**Fig. 2:**
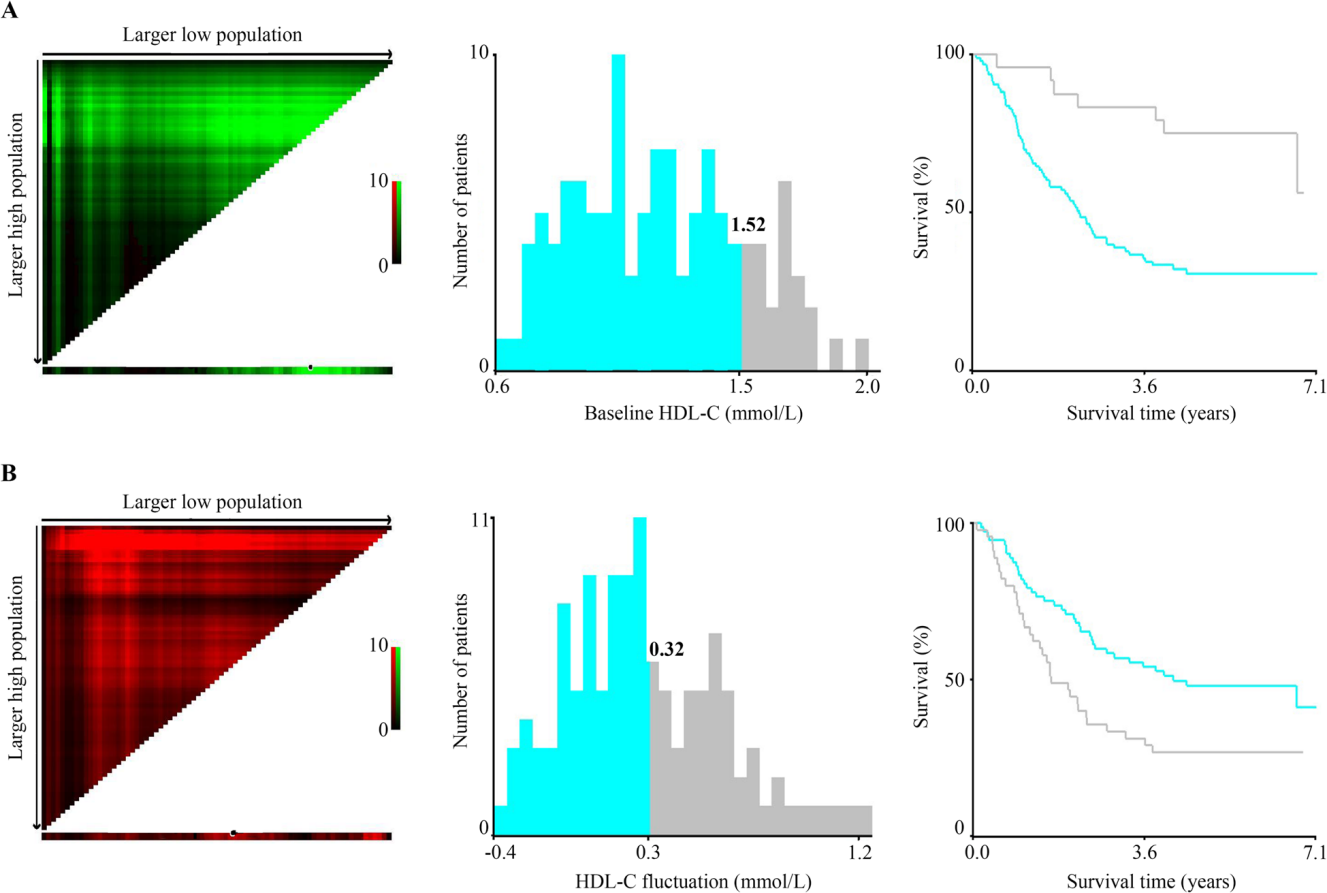
X-tile analyses stratified by the optimal HDL-C cut-off value at baseline and fluctuation. The X-tile plots in the left panels show the χ2 log-rank values, which represent all possible divisions of the lipid levels into low or high. The X-axis represents a low population, while the Y-axis represents a high population. The optimal cut-off values are highlighted by the black circles in the left panels and are shown in the histograms (the middle panels) and Kaplan–Meier plots (the right panels). **A** The optimal cut-off value for baseline HDL-C was 1.52 mmol/L. **B** The optimal cut-off value for HDL-C fluctuation was 0.32 mmol/L

X-tile was used to find the optimal cutoff value for the serum lipids based on DFS. The cutoff for the HDL-C serum level before adjuvant chemotherapy was 1.52 mmol/L. Using this value, patients were stratified as having a high risk of disease recurrence (Fig. 2A). The optimal cutoff value for cholesterol, TG, LDL-C, ApoA-I, and ApoB, were 6.12 mmol/L, 1.36 mmol/L, 2.08 mmol/L, 0.85 g/L, and 0.74 g/L, respectively.

### Lipid-related survival estimates for DFS

**Fig. 3:**
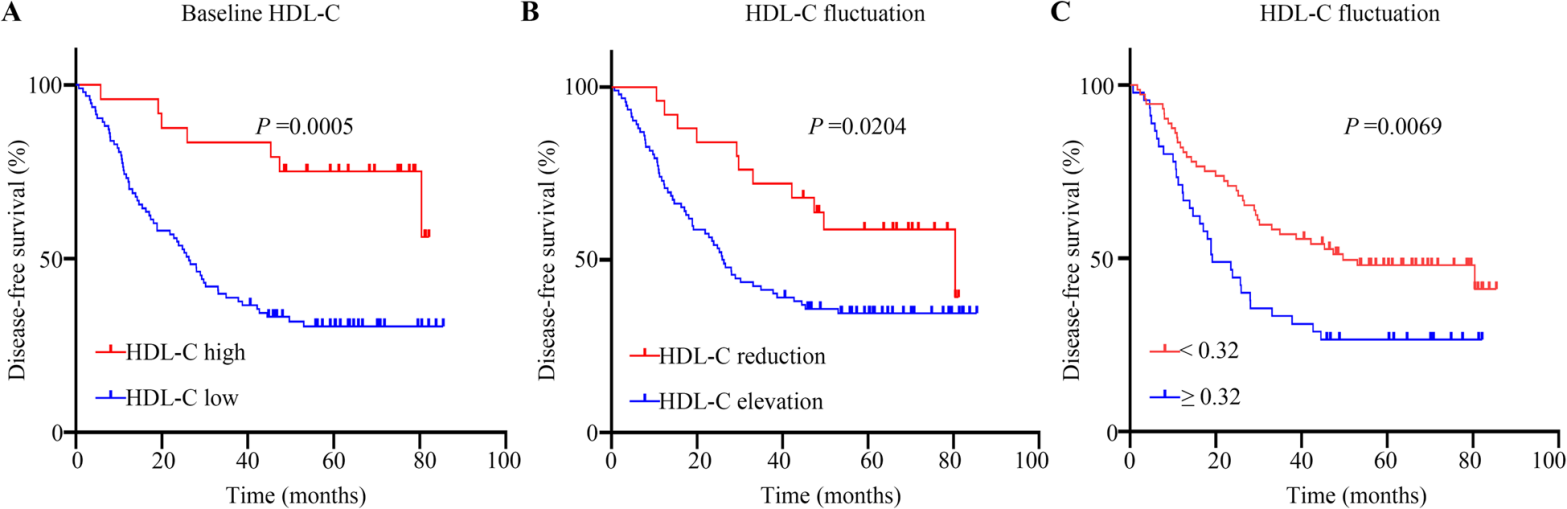
Survival curves. **A**: DFS differed significantly between patients with a serum HDL-C level at baseline ≥ 1.52 mmol/L and those with a serum HDL-C level at baseline < 1.52 mmol/L (median DFS: not reached vs. 26.30 months, *P* = 0.0005). **B**: DFS differed significantly between patients with an increased HDL-C and those with a decreased HDL-C after adjuvant chemotherapy compared with baseline (median DFS: 26.12 vs. 80.43 months, *P* = 0.0204). **C**: DFS differed significantly between patients with a serum HDL-C level that increased by ≥ 0.32 mmol/L and those with a serum HDL-C level that increased by < 0.32 mmol/L (median DFS: 19.17 vs. 49.70 months, *P* = 0.0069)

Serum lipid and lipoprotein levels before adjuvant chemotherapy were categorized as “high” or “low” based on the cut-offs determined by X-tile (Table 3). The relationship between the lipid levels and DFS for NSCLC patients were analyzed. The median DFS for patients with high HDL-C was unavailable, as only seven out of 24 patients reached the endpoints, while patients with low HDL-C had a median DFS of 26.30 months (range, 0.70–85.47). High baseline HDL-C was associated with a superior DFS (*P* = 0.0005) (Fig. 3A and Table 3). The median DFS for patients with high ApoB and those with low ApoB was 37.80 months and 11.83 months, respectively (*P* = 0.0316) (Table 3). There were no statistical differences between the high and low cohort for cholesterol, triglyceride, LDL-C or ApoA-I.

**Table 3:** Baseline lipids for DFS

Lipids	N (%)	Number of events	DFS (months), median and range	*P* value
**Cholesterol**^a^				0.132
High	105 (89.7)	66	80.43 (7.80-81.17)	
Low	12 (10.3)	5	29.33 (0.70-85.47)	
**Triglyceride**^a^				0.0529
High	34 (29.1)	25	25.05 (1.70–83.8)	
Low	83 (70.9)	46	44.50 (0.70-85.47)	
**HDL-C**^a^				**0.0005**
High	24 (20.5)	7	Unmature (5.83–82.13)	
Low	93 (79.5)	64	26.30 (0.70-85.47)	
**LDL-C**^a^				0.0520
High	101 (86.3)	59	37.80 (0.70-85.47)	
Low	16 (13.7)	12	14.75 (3.23–64.63)	
**ApoA-I**^a^				0.252
High	102 (87.2)	65	30.20 (0.70-82.33)	
Low	15 (12.8)	6	Unmature (4.60-85.47)	
**ApoB**^a^				**0.0316**
High	103 (88.0)	61	37.80 (0.70-85.47)	
Low	14 (12.0)	11	11.83 (3.23–63.37)	

Abbreviations: *DFS *disease free survival, *HDL-C *high-density lipoprotein cholesterol, *LDL-C *low-density lipoprotein cholesterol, *ApoA-I *apolipoprotein A-I, *ApoB *apolipoprotein B

^a^Comparison between baseline lipids of high and low

Correlations between serum lipid fluctuations and survival were analyzed (Tables 4 and 5). Patients with a serum HDL-C level that decreased after adjuvant chemotherapy exhibited superior DFS compared with patients with an increased level (*P* = 0.0204). The median DFS for patients with elevated HDL-C levels was 26.12 (range, 0.70–85.47) months, while the median DFS for patients with decreased HDL-C levels was 80.43 (range, 10.53–81.17) months (Table 4, and Fig. 3B). Specifically, the optimal cut-off value of lipid fluctuation after adjuvant chemotherapy was determined using X-tile (Fig. 2B). The results suggested that NSCLC patients with a serum HDL-C level that increased by 0.32 mmol/L or above after chemotherapy had an inferior DFS (HDL-C fluctuation < 0.32 vs. ≥0.32mmol/L: median DFS: 49.70 vs. 19.17 months, *P* = 0.0069) (Table 5, and Fig. 3C). Moreover, a triglyceride increase ≥ 0.25 mmol/L and an increase in ApoA-I ≥ 0.50 g/L indicated a poorer DFS (Table 5).

**Table 4:** Lipid alterations for DFS

Lipids	N (%)	Number of events	DFS (months), median and range	*P* value
**Cholesterol**^a^				0.775
Elevation	93 (79.5)	55	33.10 (1.70-85.47)	
Reduction	24 (20.5)	16	34.47 (0.70-81.17)	
**Triglyceride**^a^				0.949
Elevation	82 (70.1)	50	34.03 (0.70-85.47)	
Reduction	35 (29.9)	21	30.20 (3.23–81.37)	
**HDL-C**^a^				**0.0204**
Elevation	92 (78.6)	60	26.12 (0.70-85.47)	
Reduction	25 (21.4)	11	80.43 (10.53–81.17)	
**LDL-C**^a^				0.919
Elevation	84 (71.8)	50	31.22 (1.70-85.47)	
Reduction	33 (28.2)	21	34.97 (0.70-81.17)	
**ApoA-I**^a^				0.286
Elevation	97 (82.9)	61	28.97 (0.70-85.47)	
Reduction	20 (17.1)	10	80.43 (1.70-81.17)	
**ApoB**^a^				0.887
Elevation	81 (69.2)	49	33.10 (1.70-85.47)	
Reduction	36 (30.8)	22	34.47 (0.70-81.17)	

Data are mean ± standard deviations

Abbreviations: *DFS *disease free survival, *HDL-C *high-density lipoprotein cholesterol, *LDL-C *low-density lipoprotein cholesterol, *ApoA-I *apolipoprotein A-I, *ApoB *apolipoprotein B

^a^Comparison between lipids post-chemotherapy and lipids pre-chemotherapy

**Table 5:** Optimal cut-off values of lipid fluctuation for DFS

Lipid fluctuation^a^	N (%)	Number of events	DFS (months), median and range	*P* value
**Cholesterol** (mmol/L)				0.20
< 0.14	16 (13.7)	8	80.43 (0.70-81.17)	
≥ 0.14	101 (86.3)	63	28.97 (1.70-85.47)	
**Triglyceride** (mmol/L)				**0.020**
< 0.25	61 (52.1)	31	47.40 (3.23–85.47)	
≥ 0.25	56 (47.9)	40	26.28 (0.70–83.80)	
**HDL-C** (mmol/L)				**0.0069**
< 0.32	72 (61.5)	38	49.70 (1.70-85.47)	
≥ 0.32	45 (38.5)	33	19.17 (0.70-82.13)	
**LDL-C** (mmol/L)				0.17
< 1.15	91 (77.8)	59	29.73 (0.70-82.33)	
≥ 1.15	26 (22.2)	12	Unmature (3.23–85.47)	
**ApoA-I** (g/L)				**0.018**
< 0.50	90 (76.9)	50	42.17 (0.70-85.47)	
≥ 0.50	27 (23.1)	21	18.93 (4.60–77.60)	
**ApoB** (g/L)				0.14
< 0.14	72 (61.5)	48	27.32 (0.70-82.07)	
≥ 0.14	45 (38.5)	23	53.00 (2.37–85.47)	

Data are mean ± standard deviations

Abbreviations: *DFS *disease free survival, *HDL-C *high-density lipoprotein cholesterol, *LDL-C *low-density lipoprotein cholesterol, *ApoA-I*  apolipoprotein A-I, *ApoB *apolipoprotein B

^a^Difference = lipids post-chemotherapy –lipids pre-chemotherapy

### Prognostic indicators for NSCLC after chemotherapy

Whether serum lipid and lipoprotein levels (or fluctuations in their levels) during chemotherapy could predict DFS was explored for stage IB–IIIA NSCLC patients undergoing radical resection and adjuvant chemotherapy. Serum lipid and lipoprotein levels before chemotherapy were classified as high or low, lipid profile chemotherapy-related fluctuations, and other clinicopathological characteristics were included in the univariate and multivariate analyses (Table 6).

**Table 6:** Predictive factors for DFS by univariate and multivariate analysis

		Univariate analyses		Multivariate analyses	
		**HR (95 % CI)**	***P*value**	**HR (95 % CI)**	***P*value**
**Gender**	male vs. female	0.954 (0.594–1.533)	0.847		
**Year**	< 65 vs. ≥65	1.035 (0.740–1.447)	0.842		
**BMI** (kg/m^2^)	< 24 vs. ≥24	1.026 (0.624–1.699)	0.918		
**Histology**	adenocarcinoma vs. non-adenocarcinoma	0.944 (0.553–1.612)	0.833		
**Disease Stage**	I-II vs. III	1.011 (0.633–1.613)	0.965		
**Smoking status**	smokers vs. non-smokers	1.656 (1.022–2.685)	**0.041**	1.025 (0.575–1.830)	0.932
**Drinking status**	non-drinkers vs. drinkers	1.219 (0.705–2.106)	0.478		
**Surgical type**	lobectomy vs. pneumonectomy	2.062 (0.648–6.564)	0.221		
**T stage**	1–2 vs. 3–4	0.567 (0.311–1.035)	**0.054**	0.719 (0.384–1.345)	0.302
** N stage**	0–1 vs. 2	0.329 (0.202–0.534)	**< 0.001**	0.353 (0.203–0.612)	**< 0.001**
**Radiotherapy**	no vs. yes	0.727 (0.348–1.521)	0.398		
**Baseline cholesterol**	low vs. high	1.986 (0.799–4.938)	**0.140**	1.449 (0.508–4.130)	0.488
**Baseline triglyceride**	low vs. high	0.620 (0.380–1.011)	**0.055**	0.652 (0.385–1.103)	0.111
**Baseline HDL-C**	low vs. high	2.702 (1.291–5.652)	**0.008**	1.717 (0.735–4.014)	**0.021**
**Baseline LDL-C**	low vs. high	1.837 (0.985–3.427)	**0.056**	0.803 (0.386–1.671)	0.557
**Baseline ApoA-I**	low vs. high	0.616 (0.266–1.423)	0.256		
**Baseline ApoB**	low vs. high	2.047 (1.073–3.905)	**0.030**	2.100 (0.999–4.417)	0.050
**Cholesterol fluctuation**	reduction vs. elevation	1.085 (0.622–1.893)	0.775		
**Triglyceride fluctuation**	reduction vs. elevation	0.984 (0.591–1.638)	0.949		
**HDL-C fluctuation**	reduction vs. elevation	0.475 (0.249–0.904)	**0.023**	0.609 (0.310–1.200)	**0.015**
**LDL-C fluctuation**	reduction vs. elevation	1.027 (0.616–1.711)	0.919		
**ApoA-I fluctuation**	reduction vs. elevation	0.696 (0.356–1.359)	0.289		
**ApoB fluctuation**	reduction vs. elevation	1.037 (0.627–1.717)	0.887		

Data are mean ± standard deviations

Abbreviations: *DFS *disease free survival, *BMI *Body Mass Index, *HDL-C *high-density lipoprotein cholesterol, *LDL-C *low-density lipoprotein cholesterol, *ApoA-I*  apolipoprotein A-I, *ApoB *apolipoprotein B, *HR *Hazard ratio, *CI *Confidence interval

In the univariate analyses, a chemotherapy-related HDL-C reduction exhibited superior DFS (hazard ratio [HR]: 0.475; 95 % confidence interval [CI]: 0.249–0.904, *P* = 0.023). In the multivariate analyses, a chemotherapy-related HDL-C reduction independently predicted DFS (HR: 0.609; 95 %CI: 0.310–1.200, *P* = 0.015). A high baseline HDL-C level also independently indicated superior DFS (low vs. high: HR: 1.717; 95 %CI: 0.735–4.014, *P* = 0.021). N stage was found to be an inferior independent indicator for DFS. In the univariate analyses, N stage was associated with DFS (N0-1 vs. N2: HR: 0.329; 95 %CI: 0.202–0.534, *P* < 0.001). In the multivariate Cox regression analyses, N stage was predictive of DFS (N0-1 vs. N2: HR: 0.353; 95 %CI: 0.203–0.612, *P* < 0.001). And a low baseline ApoB and drinking had a negative effect on DFS in the univariate analysis (Table 6).

### Correlations between HDL-C and clinical characteristics

A number of correlations between HDL-C and clinical characteristics were observed: (1) a high baseline HDL-C was related to N0-1 stage (χ^2^ = 6.413, *P* = 0.011), (2) non-smokers (χ^2^ = 6.106, *P* = 0.013) and chemotherapy other than pemetrexed-based regimens (χ^2^ = 5.002, *P* = 0.025) were prone to a HDL-C reduction, and (3) no association was found between HDL-C level and the other parameters, including gender, BMI, stage or surgical type (Table 7).

**Table 7:** Relationships between HDL-C and clinical data

	Baseline HDL-C^a^			HDL-C fluctuation^b^		
**Characteristics**	**High**	**Low**	***P*Value**	**Elevation**	**Reduction**	***P*Value**
**Age, n (%)**						
< 65	16 (13.7)	83 (70.9)	0.086	77 (65.8)	22 (18.8)	0.597
≥ 65	6 (5.1)	12 (10.3)		15 (12.8)	3 (2.6)	
**Gender, n (%)**						
Female	11 (9.4)	36 (30.8)	0.297	40 (34.2)	7 (6.0)	0.162
Male	11 (9.4)	59 (50.4)		52 (44.4)	18 (15.4)	
**BMI (kg/m**^**2**^**), n (%)**						
< 24	16 (13.7)	63 (53.8)	0.563	60 (51.3)	19 (16.2)	0.307
≥ 24	6 (5.1)	32 (27.4)		32 (27.4)	6 (5.1)	
**Smoking status, n (%)**						
Non-smoker	8 (6.8)	55 (47.0)	0.068	55 (47.0)	8 (6.8)	**0.013**
Smoker	14 (12.0)	40 (34.2)		37 (31.6)	17 (14.5)	
**T stage, n (%)**						
1–2	19 (16.2)	74 (63.2)	0.375	73 (62.4)	20 (17.1)	0.943
3–4	3 (2.7)	21 (17.9)		19 (16.2)	5 (4.3)	
**N stage, n (%)**						
0–1	17 (14.5)	45 (38.5)	**0.011**	45 (38.5)	17 (14.5)	0.090
2	5 (4.3)	50 (42.7)		47 (40.2)	8 (6.8)	
**Disease stage, n (%)**						
Stage I-II	8 (6.8)	45 (38.5)	0.350	41 (35.0)	12 (10.3)	0.760
Stage III	14 (12.0)	50 (42.7)		51 (43.6)	13 (11.1)	
**Histology, n (%)**						
Adenocarcinoma	15 (12.8)	71 (60.7)	0.530	67 (57.3)	19 (16.2)	0.750
Non-adenocarcinoma	7 (6.0)	24 (20.5)		25 (21.4)	6 (5.1)	
**Drinking status, n (%)**						
Non-drinkers	14 (12.0)	71 (60.7)	0.293	68 (58.1)	17 (14.5)	0.556
Drinkers	8 (6.8)	24 (20.5)		24 (20.5)	8 (6.8)	
**Radiotherapy, n (%)**						
Non-radiotherapy	21 (17.9)	85 (72.6)	0.386	83 (70.9)	23 (19.7)	0.787
Radiotherapy	1 (1.0)	10 (8.5)		9 (7.7)	2 (1.7)	
**chemotherapy regimens, n (%)**						
Cisplatin/carboplatin/nedaplatin + pemetrexed	15 (12.8)	71 (60.7)	0.530	72 (61.5)	14 (12.0)	**0.025**
Others	7 (6.0)	24 (20.5)		20 (17.1)	11 (9.4)	
**Surgery type, n (%)**						
Lobectomy	22 (18.8)	87 (74.4)	0.158	85 (72.6)	24 (20.5)	0.526
Pneumonectomy	0 (0)	8 (6.8)		7 (6.0)	1 (0.9)	

Abbreviations: *HDL-C *high-density lipoprotein cholesterol, *BMI *Body Mass Index

^a^Comparison between baseline lipids of high and low

^b^Comparison between lipids post-chemotherapy and lipids pre-chemotherapy

## Discussion

Here, serum lipid profiles in predicting outcomes were assessed in NSCLC patients who received adjuvant chemotherapy after radical surgery. Cholesterol, LDL-C, HDL-C, ApoA-I, ApoB, and TG levels were all significantly elevated after chemotherapy. Patients with a high HDL-C, and ApoB at baseline as well as a chemotherapy-related reduction in the HDL-C level had a longer DFS. Furthermore, a reduction in the HDL-C level, and a high HDL-C level at baseline were independent predictors of a longer DFS.

Extensive studies have indicated that serum lipid levels are altered following chemotherapy. Yet, the evidence is inconsistent, with differences among various cancers. An *in vitro* study on the commonly used lung cancer drug docetaxel showed increased HDL-C biogenesis in primary human skin fibroblasts, macrophages and smooth muscle cells [24]. An earlier study on chemo-sensitive cancers also showed an increase in cholesterol and LDL-C levels after chemotherapy [25].

However, LDL-C and HDL-C can also be altered in reverse. Wang’s large cohort study of 667 colorectal cancer patients exhibited significantly increased HDL-C and decreased LDL-C after receiving fluoropyrimidine-based chemotherapy [19]. According to Li, and Arpino et al. patients with breast cancer showed significant LDL-C elevation and HDL-C reduction post-chemotherapy [15, 16]. Sharma M et al. found that specific drugs also have different effects. Doxorubicin decreased HDL-C, whereas, paclitaxel increased ApoB, yet cyclophosphamide had no effect on HDL metabolism in breast cancer patients [20]. Other agents, 5’-fluorouracil and methotrexate might also decrease HDL-C and LDL-C levels [26]. Nevertheless, this study here, is the first to report the lipid fluctuations after adjuvant chemotherapy in NSCLC. Pemetrexed-based regimens specifically, were more prone to increasing HDL-C.

There are fewer studies on chemotherapy affecting blood lipids, with most focusing on the mechanisms. Some chemotherapy drugs can affect lipid metabolism in liver cells. Doxorubicin decreases the expression of 3-hydroxy-3-methylglutaryl-coenzymeA (HMG-CoA) reductase and ATP-binding cassette transporter A1 (ABCA1) in liver cells by directly decreasing two transcription factors, the peroxisomal proliferator activated receptor γ (PPARγ) and the liver X receptor α (LXRα) [20]. In liver cells, HMG-CoA reductase mediates cholesterol synthesis and ABCA1 mediates cholesterol efflux, which are major contributors to HDL-C levels [27]. However, the effect is drug-specific, for example, cyclophosphamide, and paclitaxel do not express the same activity [20]. It has also been discovered that chemotherapy can affect system lipid metabolism on primary human skin fibroblasts, macrophages and smooth muscle cells. Desmocollin 1 (DSC1) binds to apoA-I (apoA-I-DSC1) to inhibit HDL biogenesis, whereas, docetaxel binds with high affinity as a potential inhibitor of apoA-I-DSC1 interaction [24]. These varying results on chemotherapy-related lipid alterations are probably due to the multi-faceted mechanisms that vary by cancer type and drugs. In a word, chemotherapy agents do alter lipid levels, however, accurately predicting how those levels impact cancer prognosis differs greatly.

Gender, according to Tharu BP et al., is a confounding factor. The correlation with HDL-C and gender was also analyzed in this study. Neither the baseline HDL-C nor chemotherapy-related HDL-C fluctuations were significantly different between male and females. Similarly, no association between HDL-C and gender was found in patients with gallbladder cancer [28]. However, several studies have reported higher concentrations of HDL-C in women. One gastric cancer study indicated that males had significantly lower HDL-C levels [29]. This may be attributed to sex hormone differences.

In this study, both a high baseline HDL-C and chemotherapy-related HDL-C reduction were identified to independently predict DFS for curable patients with resected NSCLC. While HDL-C often exhibits an association with tumor incidence, the results are inconsistent. HDL-C was adversely associated with a risk of breast cancer and colon cancer [30–33] but it was reported that an increased HDL-C level is related to a high risk of non-Hodgkin lymphoma [34], and colorectal cancer [19]. A lower HDL-C serum level at baseline has also been associated with tumor progression and an increase mortality among various cancers [35–37]. As for the relationship between chemotherapy-related HDL-C and tumors, patients with a high level of HDL-C before chemotherapy were reported to have a better treatment response for breast cancer [38], gastric cancer [35], and prostate cancer [39]. In breast cancer, LDL-C elevation after chemotherapy showed better clinical responses [38]. Patients with a complete remission exhibited a significant HDL-C elevation after induction treatment for ALL [21]. For small cell lung cancer patients, chemotherapy with a decreased HDL-C may predict disease progression [22], yet, chemotherapy with an elevated HDL-C indicated a better survival for colorectal cancer patients [19].

Evidence concerning the possible mechanism for the favorable influence of HDL-C on DFS have been examined in some experimental studies where HDL-C was used to induce apoptosis, generate oxidative stress, and dysregulate the inflammation system and thereby, influence carcinogenesis [40, 41]. The NSCLC patients in this study received surgical resection, and so the immune system may play an important part in the elimination of potential residual micro-metastases. The anti-tumor effect of HDL-C both in humans and *in vivo* are associated with altered immune responses [42, 43]. Speculatively, chemotherapy-related HDL-C and survival may be explained by the interaction between the cancer type, chemotherapy regimen and the immune system. However, the role of HDL-C in immunomodulation has both defensive and inflammatory effects. In cancers, HDL-C can regulate anti-tumor immunity and increase the recruitment of cytotoxic T cells, decrease the recruitment of myeloid-derived suppressor cells and promote the accumulation of M1 macrophages into the tumor environment [43, 44]. HDL-C has also been found to mediate an immune-suppressive effect in some non-cancerous diseases. HDL‐C increased T regulatory cells to attenuate the inflammatory process by promoting the free cholesterol efflux [45], and *in vitro*, HDL‐C promoted an anti‐inflammatory M2 phenotype [46]. M2 macrophages may stimulate tumor relapse after chemotherapy [47], with a lower level been shown to predict better prognosis for pancreatic cancer patients after chemotherapy [48].

As the recommended adjuvant treatment for NSCLC patients is cisplatin or carboplatin, it may increase M2 macrophages induced by IL10 secreted from tumor cells [49]. It is speculated that a reduction in the HDL-C level after chemotherapy might reverse the chemotherapy-induced shift of M2 macrophages. This may explain why a reduction in the HDL-C level after chemotherapy is significantly correlated with longer DFS. However, further studies are needed to clarify the specific molecular mechanism between chemotherapy-related HDL-C and survival.

### Comparisons with other studies and what does the current work add to the existing knowledge

Lipids are involved in tumor growth, immunomodulation and treatment response. Serum lipid levels also change during anti-cancer therapy. It was reported that cytotoxic drugs affect blood lipid metabolism differently [20]. Breast cancer patients often display increased total cholesterol, LDL-C, and TG levels during chemotherapy [15–18], while colorectal cancer patients treated with chemotherapy show increased HDL-C and decreased LDL-C [19]. However, it remains unclear whether lipid alterations exist in NSCLC patients receiving standard adjuvant chemotherapy. Here, elevation in lipid profiles was observed during adjuvant chemotherapy in patients with NSCLC. Moreover, associations between fluctuating lipid levels and the risk of cancer progression have been studied in only a few cancers including gastric cancer, ovarian cancer and small-cell lung cancer [19, 22, 36, 37]. One common observation among these cancers was that a lower HDL-C serum level at baseline was associated with an increase in cancer progression and mortality [22, 35–37], yet, colorectal cancer patients showed a superior outcome with a chemotherapy-related elevated HDL-C [19]. The associations between fluctuating serum lipids and the clinical outcome of curable NSCLC are unknown.

In this study, relationships between chemotherapy-related lipid alterations and the survival of NSCLC patients were identified. Notably, this is the first study concerning chemotherapy-associated blood lipid fluctuations and its relationship with clinical outcomes for NSCLC patients. According to this study, a lower baseline HDL level and an increased HDL level after adjuvant chemotherapy are independent prognostic indicators for disease-free survival in NSCLC patients. In support of these findings, a recent exploratory analysis of 851 cases with colorectal cancer demonstrated that chemotherapy-related HDL-C elevation was determined to be an independent prognostic indicator for superior DFS [19]. Specifically, this study determined 0.32 mmol/L as the optimal cut-off value for HDL-C fluctuation after chemotherapy, with an HDL-C fluctuation < 0.32mmol/L indicating a better DFS. No previous study has reported a specific lipid fluctuation cut-off value for the survival of cancer patients. Compared with other available diagnostic methods, the serum HDL test is fast, repeatable, inexpensive, and simple to carry out, and it would enable more patients to monitor recurrence. These results hint that simple HDL-C fluctuation may be a useful future prognostic marker for NSCLC.

### Strengths and limitations

This is the first study to investigate the association between serum lipids and DFS in NSCLC patients treated by radical resection and adjuvant chemotherapy. The potential bias caused by the confounding factors were minimized, which improved the reliability of the findings. However, there are some limitations: (1) it was a retrospective study; (2) patients were pooled from a single center; (3) the study cohort was small; (4) we did not have OS data; and (5) lipid and lipoprotein levels were stratified in a simple dichotomous manner, more precise stratification might have affected the prognostic efficacy.

## Conclusions

This is the first study to determine the relationship between serum lipid profiles and DFS in NSCLC patients treated by radical resection and adjuvant chemotherapy. Chemotherapy significantly increased the levels of various lipids and lipoproteins. Patients with a higher level at baseline of HDL-C and ApoB as well as a reduction in the HDL-C level after chemotherapy tended to have a longer DFS. Serum HDL-C levels that increased by 0.32 mmol/L or above after chemotherapy had an inferior DFS. A chemotherapy-associated reduction in HDL-C was a favorable prognostic indicator for DFS, and may have predictive value in an adjuvant setting. Therefore, the monitoring and regulation of serum lipid profiles are of great value for lung cancer patients receiving adjuvant chemotherapy. Clinically, it may be possible to predict survival by observing fluctuations in serum lipid levels. In the future, it is anticipated that it will be a reliable indicator in predicting lung cancer prognosis. This study suggests that serum lipids should be under long-term monitoring for cancer management.

## Data Availability

The datasets used and analysed during the current study are available from the corresponding author on reasonable request. The authenticity of this article has been validated with key raw data uploaded onto the Research Data Deposit (RDD)public platform (https:// www. researchdata. org. cn). The approval RDD number is RDDA2021002116.

## Abbreviations

LC: Lung cancer
NSCLC: Non-small-cell lung cancer
LDL-C: Low-density lipoprotein-cholesterol
HDL-C: High-density lipoprotein-cholesterol
Apo: Apolipoprotein
TG: Triglyceride
DFS: Disease-free survival
ALT: Alanine aminotransferase
AST: Aspartate aminotransferase
T-BIL: Total bilirubin
ALL: Acute lymphoblastic leukemia
SYSUCC: Sun Yat-sen University Cancer Center
SD: Standard deviation
HR: Hazard ratio
CI: Confidence interval
HMG-CoA: 3-hydroxy-3-methylglutaryl-coenzymeA
ABCA1: ATP-binding cassette transporter A1
PPARγ: Peroxisomal proliferator activated receptor γ
LXRα: Liver X receptor α
DSC1: Desmocollin 1
